# Navigating Cultural Adaptation: Refugee Parents’ Perspectives on the SafeCare Parenting Program

**DOI:** 10.1007/s12134-025-01299-1

**Published:** 2025-07-28

**Authors:** Nikita Rao, Daniel J. Whitaker, Cathleen E. Willging, Erin A. Weeks, Shannon Self-Brown, Jessica Koreis, Mary Helen O’Connor

**Affiliations:** 1 Mark Chaffin Centers for Healthy Development, School of Public Health, Georgia State University, P.O Box 3995, Atlanta, GA 30303 USA; 2 Pacific Institute for Research and Evaluation, 851 University Blvd SE, Suite 101, Albuquerque, NM 87106 USA; 3 Social Ecology and Adolescent Development Laboratory, Urban Life Building, Room 1113, 10 Decatur Street, Atlanta, GA 30303 USA; 4 Center for Community Engagement, Georgia State University, 555 N. Indian Creek, Clarkston, GA 30021 USA

**Keywords:** Adaptation, Qualitative research, Refugee families, Parenting interventions

## Abstract

As the number of forcibly displaced persons worldwide increases, the number of children either forcibly displaced or born as refugees also multiplies. In addition to struggling with basic needs upon resettlement, disruption of daily life can lead to numerous stressors for forcibly displaced parents that may cause friction in the relationship between parent and child. Evidence-based parenting programs may help forcibly displaced parents reconceptualize parenting upon resettlement, strengthen the parent–child bond, and promote positive biopsychological outcomes for children. This study aims to understand refugee parents’ experience with a culturally adapted, evidence-based positive parenting program called SafeCare. We conducted semi-structured interviews with 18 refugee parents of Afghan, Burmese, or Congolese descent who completed SafeCare. Open-ended questions focused on understanding parents’ experiences, likes, and dislikes of the adapted curriculum. We applied thematic analysis to analyze the data. The authors identified six themes related to the experiences of refugee parents: expectations and reasons for engaging with SafeCare, guidance for program implementation, learning active parenting, physical learning materials that help parents acquire new skills, the utility of in-person and virtual learning methods, and provision of tailored support for navigating cultural differences. These findings demonstrate success in adapting parenting programs to a refugee context and suggest that delivering such educational programs for forcibly displaced parents may result in better outcomes for both parent and child.

**Supplementary Information:**

The online version contains supplementary material available at 10.1007/s12134-025-01299-1.

## Introduction

As of May 2024, the global number of forcibly displaced persons has reached a record 120 million (European Civil Protection & Humanitarian Aid Operations, 2024), reflecting a distressing trend of annual increases over the past 12 years. Key events in 2023, such as internal conflict within Sudan, Afghanistan, and the Democratic Republic of the Congo (DRC), clashes in the Gaza Strip in the State of Palestine, escalating violence within Myanmar, Haiti, and Somalia, and displacement due to war in Ukraine (UNHCR, 2023) have significantly contributed to this rise. Consequently, the number of children forcibly displaced from their homes has doubled between 2010 and 2022 (UNICEF, 2023). About 40% of people living in forced displacement are children, and approximately 1.9 million of those children were born as refugees (UNICEF, 2024).

Forced displacement affects individuals physically, mentally, and emotionally, impacting both parents and their children and straining their relationships (Bernhardt et al., 2024; George & Jettner, 2016; Sangalang et al., 2019). Displacement may contribute to both pre-and-post-migratory stressors, including experiencing material deprivation, natural disasters, persecution, discrimination, and isolation (Wu et al., 2021). Resettled parents face additional challenges, such as adjusting to new cultural norms and legal systems and losing social and familial support (Lewig et al., 2010; Rao et al., 2023), which can further affect parent–child dynamics.

Parenting practices among resettled refugee families are often shaped by the interaction of traditional cultural values and the demands of adapting to a new environment. For example, resettled Afghan parents often struggle to maintain traditional parenting practices in a new context where gender roles and child-rearing practices may differ drastically (Rosenberg et al., 2022). Mothers particularly face stress balancing cultural expectations with the demands of resettlement (Rosenberg, et al., 2022). Forced displacement further strains family dynamics, with adolescents experiencing identity confusion between acculturation and cultural heritage, which heightens tensions (Salley, 2024).

Burmese refugee families, particularly those from Chin and Karen ethnic groups, often experience psychological and relational strains stemming from war-related trauma and the stress of adapting to a new environment. High psychological distress exacerbated by resettlement is closely linked to personal and familial wellbeing among Chin refugee parents (Tonsing, 2020). For Karen caregivers, challenges with emotional regulation, discipline, and child compliance are compounded by war trauma and acculturative stress (Ballard et al., 2020).

Similarly, Congolese refugee families also face unique challenges in parenting after resettlement. Many Congolese refugee women, affected by and fleeing from conflict in the DRC, struggle to preserve cultural traditions amid resettlement stresses in the U.S. (Spates et al., 2024). Parenting practices are central to cultural identity, such as disciplining children and fostering family cohesion, often clash with acculturation pressures (Kirkland et al., 2022). These stresses, compounded by mental health challenges and shifting caregiving practices, affect family dynamics (Bader et al., 2020).

While Afghan, Burmese, and Congolese families face distinct challenges upon resettlement, they share experiences of cultural dislocation and disrupted family roles due to forced displacement. Addressing these issues is essential for supportive parent–child relationships, fundamental for health childhood development and well-being.

### Importance of the Parent–Child Relationship and Programs to Enhance Positive Parenting

The parent–child relationship is crucial for early childhood development and has lasting impacts on adulthood. Several seminal socioecological theories point to parents’ role in children’s development. Social Learning Theory posits that children learn emotional expression and regulation by observing their parents’ behavior (Bandura, 1977). Baumrind (1966) describes how parenting styles affect childhood cognitive development, identifying positive relationships and strong parent–child bonds as essential for greater self-efficacy and better cognitive outcomes. Additionally, Vygotski’s Sociocultural Theory of Development (1929) and Bronfenbrenner’s ecological systems theory (1994) analyze how children engage with social and structural forces in their environment. These theories are particularly relevant for children forced into migration, whose development is disrupted by abrupt transitions to new and unfamiliar environments (Donà & Veale, 2024). Positive parenting programs are widely recommended for parents and children facing difficulties with their identities and relationships post-resettlement to establish stronger parent–child bonds and promote healthier outcomes (Jeong, et al., 2021; Zhang et al., 2021).

Positive parenting practices foster warm and nurturing parent–child relationships and teach parents to use non-coercive child management skills, while maintaining structure and boundaries for their children’s healthy cognitive and physical development (Rodrigo et al., 2012). Behaviorally based parenting programs, such as SafeCare (Whitaker et al., 2021), parent–child interaction therapy (Eyberg, 1988), and Triple P (Sanders et al., 2014), focus on enhancing parent–child interactions and behavior management to engage children and prevent misbehavior. These interventions improve parental emotional, mental, and cognitive well-being, thus parental self-efficacy and parenting practices, and strengthen parent–child relationships and child health outcomes (Carr et al., 2021; Jeong et al., 2021). Such programs have been designed for various populations, including first-time mothers (Alves et al., 2024; Olds, 2006), those facing intimate partner violence (Easterbrooks et al., 2017), and parents of children with special needs (Beckers et al., 2020).

The SafeCare parenting model was initially developed as an in-home intervention for caregivers of children aged birth through five who are either at risk of or have a history of child maltreatment, including physical abuse and neglect (Gershater-Molko et al., 2003). SafeCare is an 18-session intervention that includes three modules that can be delivered in any order: positive parent–child interaction, home safety, and child health (Gershater-Molko, et al., 2003). Here, the focus is only on parent–child interaction or PCI, module. PCI teaches parents positive interaction skills in play and daily activities, and skills to structure daily activities in a way that engages children and prevents misbehaviors. Parents are taught to use positive language, touch, and labeled praise, and they are taught to explain rules and consequences, ignore minor misbehavior, and provide reinforcers for positive behaviors. Evidence indicates that SafeCare improves parenting skills (Whitaker et al., 2021) and reduces child welfare system reports (Chaffin et al., 2012). Studies testing only the PCI module have found positive impacts on parenting skills and child behaviors (Carta et al., 2013). The model has shown effectiveness across diverse family settings (Chaffin, Bard et al., 2012; Rogers-Brown et al., 2020) with little variation in outcomes, suggesting that SafeCare can improve parenting skills for a range of families.

### Parenting Interventions with Displaced Families

Parenting interventions for forcibly displaced families can improve parenting self-efficacy (Kaiser, 2022), behaviors (Lee & Kim, 2022), mental health (Osman et al., 2017), and child psychosocial and developmental outcomes (Gilliespie et al., 2022). However, the effectiveness of these programs hinges on their cultural relevance. To ensure this, most curricula are adapted before delivery and the suitability of exiting implementation models is evaluated for their target populations (Whitaker et al., 2021). These adaptations may include language adaptations, surface adaptations that make examples more relevant, and “deep” adaptations where cultural norms and practices are woven into the intervention and change mechanisms (Resnicow et al., 1999). The application of interventions to new populations has been described as a process of “scaling out,” stressing the importance of carefully considered adaptions during this process (Aarons et al., 2012). The need for adaptation prompted the modification of the SafeCare parenting program to assist refugees in adjusting to cultural norms post-resettlement.

The parent–child interaction (PCI) module of SafeCare (SafeCare-PCI) was culturally adapted for Afghan, Burmese (refugees from Myanmar who self-identified as “Burmese” in our study, including various ethnic groups such as Karen, Chin, and others), and Congolese refugee families resettled in Clarkston, Georgia (Whitaker et al., 2021). This adaptation utilized Barrera and Castro’s Heuristic Framework (2006) and Aaron’s Dynamic Adaptation Process (2012). Barrera and Castro’s framework involved iterative feedback and community engagement through five phases of information gathering, preliminary adaptations, and adaptation refinement (Barrera & Castro, 2006). Similarly, the Dynamic Adaptation Process consisted of four sequential and iterative phases incorporating client, coach, and partner feedback (Aarons et al., 2012). These phases included needs assessment, preparing adaptations, implementing changes, and ongoing feedback for ad hoc changes (Self-Brown et al., 2022).

Further content adaptations were made through community engagement with feedback from administrators, supervisors, and service providers (Self-Brown et al., 2022) to enhance both process adaptation (presentation of materials) and content adaptation (relevance of materials), ensuring cultural appropriateness. For example, feedback from Afghan, Burmese, and Congolese community members helped tailor examples, metaphors, and practices to align with their cultural norms and values, yielding a single adapted curriculum to address needs of all three communities. Additionally, after cultural adaptations were made, SafeCare-PCI learning materials were professionally translated and back-translated into six languages including those spoken by the target refugee groups, and community members reviewed the translations for accuracy and cultural relevance.

Following translations and adaptations, SafeCare-PCI was delivered in one of two ways for the purposes of a funded research trial: (1) standard implementation carried out by three refugee resettlement agencies, with services provided by trained current employees, or (2) task-shifted implementation conducted by Community Health Workers (CHWs) from the ethnic communities being served (Whitaker et al., 2021). The outcomes of the initial study are currently under analysis and will be addressed in future work upon completion of further evaluation. These outcomes are not the primary focus of the current paper.

The current study presents qualitative data from a sub-sample of refugee parents who participated in the aforementioned trial to explore their experiences with SafeCare’s culturally adapted PCI module. This module emphasizes positive parenting skills, such as stimulating play activities, positive verbalizations, and structured activities (Lutzker & Rice, 1984; Sanders et al., 2014) and aims to enhance parent–child communication and bonding while also teaching key behavior management skills, such as planning, instructions, child engagement, and reinforcing good behavior. The objective of this study is to examine the perspectives of 18 refugee parents on the impact of SafeCare-PCI and to assess the effectiveness of its cultural adaptation.

## Methods

### Participants

Our paper presents data from a larger project that adapted the SafeCare parenting program for Afghan, Burmese, and Congolese refugees resettled in Clarkston, Georgia. These groups were selected because they represent large and distinct ethnic communities within the refugee population in the area. Participants were selected from mothers who enrolled in a research study in which they received the six-session PCI module of SafeCare and completed three quantitative surveys (pre-, post-, and 3-month follow-up). Eligibility was restricted to parents who reported speaking English *Well* or *Very Well* on the baseline survey (note: the module and surveys were administered in the participant’s native language. In cases where a survey had not been translated, it was collected in English). Parents who did not speak English proficiently were excluded from participation in this qualitative study due to resource constraints, interviews were conducted in English. Two female graduate research assistants, one Asian and one White, conducted qualitative semi-structured interviews with these parents about their SafeCare-PCI experiences in English between July 1 st, 2022, and April 26th, 2024. Of the 18 interviews, one was excluded due to technical issues, resulting in a final sample of 17 females: six each from the Afghan and Burmese communities and five from the Congolese community. Participants averaged 29.3 years of age (range: 21.6 to 35.5 years), had 2.33 children on average, and had lived in the U.S. an average of 7.75 years. Demographic characteristics of participants are further presented in Table 1 (Demographic Profile of Refugee Parents Participating in the SafeCare Program).

**Table 1:** Demographic profile of refugee parents participating in the SafeCare Program^a^

**Variable**		*N* (%) or M (SD)
Ethnicity		
	*Afghan*	6 (38)
	*Burmese*	5(31)
	*Congolese*	5 (31)
Parent age		29.32 (3.80)
Target child age in years		2.33 (1.36)
Total children		2.25(1.53)
Married		12 (92)
Working full-or-part-time		3 (31)
High school diploma or higher		16 (100)
Education completed in United States		6 (38)
Currently in school		2 (13)
English fluency		
	*Not well*	1 (6)
	*Well*	8 (50)
	*Very well*	7 (44)
Annual income		
< *$15,000*	< *$15,000*	3 (23)
*$15,000–$24,999*	*$15,000–$24,999*	4 (31)
> *$25,000*	> *$25,000*	6 (46)
Years in birth country		18.63 (8.51)
Years in refugee camp		5.31 (6.31)
Countries lived in		2.31 (0.70)
Years in United States		7.75 (5.07)

^1^Parent age, target child age, total children, years in birth country, years in refugee camp, countries lived in, and years in USA are presented as means (SD). All others are numbers and percents

### Procedure

Participants were invited to a qualitative interview after voluntarily completing the SafeCare-PCI intervention. Interviewers read and obtained verbal, informed consent from all participants. The Georgia State University Institutional Review Board reviewed and approved the study protocol.

### Qualitative Interviews

Qualitative interviews for this paper explored parents’ views of the utility of SafeCare-PCI materials, its impact on parent–child bonding, and the parenting skills acquired. These interviews featured open-ended questions about parents’ interest in SafeCare-PCI satisfaction with providers, and skill acquisition. Trained in qualitative interviews, the research assistants probed for more specific information on program recommendations and cultural appropriateness. All interviews were conducted virtually on Zoom or Google Voice due to the COVID-19 pandemic. Interviews averaged 27.18 min (range: 14 to 45 min) and were recorded and professionally transcribed. Interviewer debriefing templates were completed after each interview to help researchers reflect on their experiences, capture insights about participant dynamics, and identify emerging themes that could inform data analysis. Participants received between $25 and $50 for their time.

We used Braun and Clarke’s (2012) thematic analysis process to identify patterns and themes in our data. First, three transcripts were randomly selected for initial coding to develop the preliminary codebook. The lead coder created this codebook through open coding based on interview questions and topics. This codebook included codes, subcodes, and definitions. Then, three coders, including the lead coder, independently coded these transcripts using the preliminary codebook. The team, two Asian females and one Asian male, reviewed and discussed coding, with only minor revisions to the codebook. The remaining 14 transcripts were divided between the coders for completion. The lead coder developed a framework matrix following the final coding to identify overarching patterns and themes. The lead coder then consulted with a qualitative expert to refine themes, explore their interrelationships, and finalize the thematic framework.

## Results

Our iterative analysis identified six interrelated themes across responses: (1) Expectations and motivations for engaging with SafeCare-PCI, (2) provider support and cultural sensitivity in implementation, (3) impact on parenting, (4) challenges and successes in SafeCare-PCI implementation, (5) delivery methods and social support, and (6) navigating cultural differences through SafeCare-PCI. Quotations in the results are presented verbatim, with filler words removed for clarity, while preserving grammar and vocabulary to minimize researcher modifications.

### Theme 1: Expectations and Motivations for Engaging with SafeCare-PCI

Many parents heard about SafeCare-PCI through their community networks or friends, highlighting the importance of social connections in facilitating participation. Parents from all three communities shared that their primary motivation for joining SafeCare-PCI was to improve their interactions with their children and strengthen their bonds. These insights illustrate that parents were motivated by a genuine commitment to their children’s well-being and development:

Yes. It’s good for them, and I learned more things about my children… how can I spend with them time, and how can I take time for my children, play with them and they play with me. I talk with them.… I enjoy the time with my children. (Afghan mother)

I like to get more experiences to help my child to develop skills that can have my child grow to be a better person.... When I heard about SafeCare, one of my friends told me about it…. I thought, “Oh, let me try how American people raise their child and compare to our culture. I want to compare the things. What’s the difference between raising a child back home and in here?” Yeah, and then I say, “Oh, maybe I get more experience, how to raise a child in here.” (Burmese mother)

Oh yeah, she [SafeCare provider] told me that SafeCare is a program that helps parents to improve on knowing better with their children, to know their development, and how we as parents, we can live with the kids. I would say that it’s more about educating parents how to live with their children and to understand them better. (Congolese mother)

Participants were incentivized after completing the full program. While participants appreciated the gift cards, they emphasized that their motivation to participate in SafeCare-PCI extended far beyond the financial incentive. Across all three cultural groups, parents expressed that the experiences and parenting skills they gained were far more valuable to them:

If there were no gift cards, I [would] still do it because I have learned a lot of things. I don't know about other people, but I learned so [much] good stuff. (Afghan mother)

And then this gift card is, I think this is just a gift.... [The program] is very helpful for parenting…. [If] you want to learn more, they will give you some method to deal with your children. (Burmese mother)

It’s not because of the money. It’s because of learning. I just receive the gift card. I can say it’s a gift and I have to take it. But it’s not like I want to do the SafeCare because they’re giving money. No, I want to do it because I want to learn more. (Congolese mother)

Participants stated they would recommend SafeCare-PCI to friends and family, highlighting its value in improving communication with and understanding their child’s needs, especially in a new country:

I would say, “You should definitely try the plan because it’s going to help you interact with your kid better, understand your kid better, and you’ll have a routine every single day.” (Burmese mother)

Yeah. If I talk to a friend [about] SafeCare… [SafeCare helps] parents how to educate their kids, how to be close to their kids. Because have parents, especially African parents, we are ignorant of everything. We always think that to correct a child, you have just to beat him, to do not give him food. That is we always think. Or, is not that. (Congolese mother)

Refugee mothers reported that SafeCare-PCI promoted closer bonds with their children and helped them understand U.S. parenting norms, which differ from those of their home country. Their willingness to recommend SafeCare-PCI to family and friends underscores its perceived effectiveness in equipping parents with essential knowledge and skills. Parents articulated a desire to share their newfound understanding with others, recognizing that such knowledge can transform parenting practices and improve the well-being of children within their cultural contexts.

### Theme 2: Provider Support and Cultural Sensitivity in Implementation

Refugee parents provided feedback on their overall experiences with SafeCare-PCI, noting aspects they liked or disliked about the program. Parents highlighted how the program’s delivery by flexible and respectful providers enhanced their learning experience. One Afghan mother shared:

I think she was a great lady [the SafeCare provider].… She was kind…and she was very flexible. She didn’t push us like, “Okay, the baby has to do this, this, this.”... If the baby is not okay with [the] activities, she didn’t try to push us. But I got the information. I got the idea.

Parents frequently mentioned that their providers were patient with them and their children, which reduced their stress and allowed them to prioritize their children’s needs. For example, one Congolese mother remarked:

I remember one day when I was doing the session, so the baby was sleeping…. And in few minutes, she just wake up. When she wake up, I have to talk to the lady [SafeCare provider]…. She was there to ask me, because the baby was crying. She was there to ask me, “Is the baby okay? Is she sick?” Or something like that. And she told me, just take a few minutes to check on her or give her something so she can calm down…. That one for me was very, very good for me. Because when I saw her, she care about my baby. I can tell my family...She [my baby] calm down, and then we continue with the session.

Additionally, parents found that the CHWs delivering SafeCare-PCI who were culturally similar or competent providers facilitated learning well, as they could communicate and connect more easily with them:

She was very considerate of my time and her time. And then the greatest with my son, we can speak to each other in our native tongue, so it’s easier for us just to go through the steps of how to do the certain things during that time. (Burmese mother)

Yes, she was [sic] too, and she knows our culture. It was respectful all the time actually to us. She was able to come at the last session with us at home and then she was great…. I didn’t see anything bad at all. Yeah. (Afghan mother)

Parents also appreciated how their providers supported virtual learning by assisting with device use and navigating Zoom for their sessions:

The experience I have is when I was trying to participate in the session, that was so hard for me to get on Zoom.... She [SafeCare provider] helped me. She called me on…my baby’s iPad. She called me on the phone and then try to explain to me, “Do this, you can click here, click here,” and boom. I just did. (Congolese mother)

There was a time I had issues with the iPad because I was using my daughter’s iPad. The background wasn’t clear, so I need to work, I have to change the iPad, and then she told me, “You have to put [it] in this position.” She was there to make sure she’s able to see me and the child and make sure we are okay. (Congolese mother)

These results offer implementation guidance for effective delivery, including strategies for dynamic settings. Parents noted that having providers familiar with their culture enhances relationship-building and facilitates learning SafeCare-PCI skills. Often, these providers were CHWs from the same or similar ethnic backgrounds as the participant, fostering trust and relatability that benefited their engagement with the program. Additionally, providers had duties beyond delivering the parenting intervention, such as helping with technology use so parents could participate in their sessions.

### Theme 3: Impact on Parenting

Upon resettlement, refugee parents faced challenges with both acculturation and parenting. They reported that SafeCare-PCI enabled them to develop active parenting skills and bond with their child. For example, some parents shared how creating a routine helped to manage their child’s behavior:

Because I told her [the Safecare provider] that I was a little bit struggling with the sleeping time at night. And then the routine was to just go and give them some time, read a book or whatever he wants. He is mostly like to have some song. We are just singing some song and then he is going to sleep easily now. (Afghan mother)

Yeah, later we did wake up together, make bed, and then we head to the bathroom and wash face, brush teeth…. And then later, when it’s time, take a snack for my daughter…. That was very helpful, …. [usually] I’ll just forget about one thing. But with the SafeCare program, I was able to follow the routine as soon as we wake up... Yeah, it was cool. (Burmese mother)

Mothers shared that allowing children to choose their activities, clothing, and toys enhanced their involvement in decision-making and strengthened their bond:

The experience overall was fantastic, I could say. The one thing that they emphasized a lot [is] to read a book with a baby and give them a right to choose. And that is when I think before I was like, whatever I want, I give it to baby and now I’m giving a right to choose between the toys, between the clothes, whatever they want. I’m going to put some things and he is able to choose the one he likes the most. And that’s [what] got from that program. (Afghan mother)

No, let me create a good communication with the baby…When she take a bath, I have to give a choice for baby. Before I was not. I was just choosing it basically myself. I give it to her but. Now, I think it, “Mommy will do it. Which kind of [inaudible] do you choose?” If they need shoes for school. “What shoes do you want to wear?” “Do you want to wear this one? What color?” Yeah, it’s so important. (Congolese mother)

Parents noted that describing upcoming activities to their child, a key SafeCare-PCI skill, helped them prepare mentally, making it easier for both parent and child to complete the tasks. For example, one Afghan mother stated:

Yes. When I bring food for my baby and I want to take food for my baby, before I bring the food, I say to my baby, I’m going to bring a food for you. I want to wash your hands. (Afghan mother)

Mothers across all cultures shared that learning and combining these skills allowed them to spend more quality time with their children and better understand their preferences, strengthening bonds:

Yes. It’s good for them and I learned more things about my children… how can I spend with them time, and how can I take time for my children, play with them and they play with me. I talk with them…. I enjoy the time with my children. (Afghan mother)

I think one of the greatest thing I liked about this program that I get to learn more about my baby, what she likes…. And then I also realize that sometimes when she plays, she needs attention from me, although she can’t speak yet, but I can tell her she needs mother to be there and to watch her play. She needs like, “Peekaboo.” She was like, that she needs me to interact with her. That’s one, another thing that I learned, mm-hmm. (Burmese mother)

Yes. The most helpful what I learn in SafeCare is playing with my kids. Before, I was not that kind of parent. No time for my kids. I know they can play themself.... But I didn’t know that I have to play with them, or there is some educative gain that’s going to help them improve them at school, improve their knowledge…. I thought that playing is playing. But since then, I started [to] teach them how we can play by writing, we can play by coloring, by painting, which is helpful at school too. (Congolese mother)

Overall, parents reported that SafeCare-PCI taught them various skills for managing their children’s behaviors in a nurturing and encouraging way. They noted that combining these skills helped them spend more time with their children, enhancing their enjoyment and understanding of their children’s needs and fostering stronger, more stable relationships.

### Theme 4: Challenges and Successes in SafeCare-PCI Implementation

SafeCare-PCI implementation includes a booklet with visual and verbal examples for interacting with children, offered in English and several native languages. While most parents found translations to be accurate and easily understood, some noted mistranslations or poor interpretations:

No, it was really correct. It was good, and everything was... Yeah, because I got the book in three languages, in English, Pashto, and Dari, and what I’m seeing is the same translation. Yeah. (Afghan mother)

They’re very understandable. Because sometimes I feel that with the translations, sometimes it’s hard to understand. But this one is very understandable. (Burmese mother)

Yeah. Yeah, it’s good. Yeah, plus, we have the Karen book. Yeah, it’s easier for me or for other moms to read. (Burmese mother)

Oh, yeah. Oh my God, I can’t even believe because in some pages that I was trying to read, it was mostly Google Translate. So mostly it was Google Translate, and that really doesn’t make sense to us. But yeah, it was mostly Google Translate I can say. I don’t know who translated, but it was very weird translations. (Afghan mother)

The translation, some words in Karen, I don’t understand. Because, and I feel like, I’m not trying to be rude to the translator, it’s just that sometimes the first thing I noticed was the “Yes” and “No” questions, right… is [is] so rude when you say that. When people talk to you, do you like this? You’re like, ‘Huh-uh’? No. That’s so rude. The differentiator, that part, yeah. And there’s some words, I don’t even understand it. (Burmese mother)

Despite contradictory feedback about language translation, parents found the examples and pictures useful for learning new skills. However, participants requested additional examples to illustrate diverse parenting methods:

Yeah, if she has more, not only one way in the bath. Actually these are playing along and then, but I’m request is bath time and eat time, the method is the same…I hope it should be different method way to parent children or to train children, may be good for us. (Congolese mother)

Thus, overall, satisfaction with the SafeCare-PCI materials was mixed. Most parents appreciated the visual examples, but feedback on verbal translations varied. However, parents requested more examples in the booklets, highlighting their role in facilitating the learning of positive parenting skills.

### Theme 5: Delivery Methods and Social Support

Parents appreciated both virtual and in-person methods for learning SafeCare-PCI. For some, virtual sessions were more accessible due to home responsibilities and the number of children. This flexibility accommodates various parenting situations and fosters a comfortable learning environment, allowing parents to fully focus on the content without added pressure:

I would say it was really nice. From Zoom or even from home, I could learn from both ways. Some moms would prefer Zoom because sometimes their homes, because you have kids and you have a lot of other work, you cannot get up and clean and stuff, so they’re very shy. I’m not saying my side, but I would think other people, they would prefer Zoom calls, and learn the same thing. Even the providers with them, or they could call Zoom. (Afghan mother)

Another parent shared that starting with in-person sessions could enhance engagement between parents and their children, providing parents opportunities for social interaction and potentially fostering a supportive network for parents and peer interactions for children:

I would recommend if they can try to have the session in person first, and then if they can bring two or three mothers together with their babies, that would be another good example because the mothers would be able to learn from each other. And the babies can learn from each other because [at] some point, one baby is not going to engage with the activities a lot. If they see another baby, they can do it because another program that we are attending, it is much effective because there was more babies with the mothers, with the fathers sometimes… I think in future, if we have this program again, it would be great if we have two, three mothers together. (Afghan mother)

These results demonstrate that parents have varying preferences for implementation methods, with some seeking more social support through in-person and group sessions. Overall, the feedback suggests that SafeCare-PCI’s adaptation for refugee families yields positive outcomes in areas such as active parenting and cultural adjustment. Nonetheless, issues like mistranslations point to areas needing improvement.

### Theme 6: Navigating Cultural Differences Through SafeCare-PCI

Mothers reported that SafeCare-PCI parenting practices differed significantly from their prior experiences, as parenting in their home country involved fewer legal and social constraints, with social networks playing a significant role in child-rearing. However, refugee parents acknowledged the value of the skills learned in SafeCare-PCI for fostering stronger bonds and adopting gentler approaches with their children:

So, you can’t leave the kids, right? Sometimes, you can’t just leave the kids and then go like to work somewhere like that. Right here it’s like you have to take the kids for daycare. But in my country, it’s different....The daycare we have is friends and neighbors…. If you don’t have anything [like] that, you going to lock the door and then just leave them in front of the door. Yes. That’s the one. We used to do that most of the time in my country. We don’t have something like a daycare. No, we don’t have that. (Congolese mother)

With the child, back home, I do get to raise a child back home, but here, we don’t usually yell at our kid, or something like that, or hit them. It’s not like you abuse your child. Sometimes, when they don’t listen, you give some kind of punishment. Sometimes, you shoot them with a rubber band or yell at them. But here is the different thing, that we don’t yell at our child or we don’t hurt them and do physical punishment. That’s the very helpful one. And then that’s the difference between our culture and the American culture. (Burmese culture)

Although parents appreciated the value of SafeCare-PCI, one Afghan mother noted that adapting to U.S. parenting norms can be challenging, particularly depending on the length of time post-resettlement, given significant cultural differences in parenting styles and expectations. She noted that the content might feel unfamiliar or strange as these practices are not commonly followed back home:

To be honest, it is because I think I would say 90% they are not practicing this curriculum, this manual in Afghanistan. Or maybe in America, I will say in America or in Canada or any other Western country, they are not practicing. Because in our culture it’s a little bit different because the direction, the treat, how they treat the baby, how they talk with the baby.... I know for other women, for other mothers, maybe this program is going to be a little bit weird because the content is not acceptable. For me, it was okay because it’s been a long time that we are here in America.

Generally, parents found SafeCare-PCI beneficial for learning U.S. parenting norms. However, many noted that SafeCare-PCI highlighted significant cultural differences in parenting compared to their home country, such as the concept of daycare, which contrasts sharply with traditional reliance on friends and family for childcare. Additionally, while mothers appreciated the emphasis on non-violent discipline, they recognized that physical punishment was common in their cultures, highlighting the complexities of reconciling new approaches with traditional beliefs.

## Discussion

This study explores the reception of the adapted SafeCare-PCI parenting program among Afghan, Burmese, and Congolese parents resettled in the U.S. As detailed in the results, participants reported favorable responses to SafeCare-PCI, with particular appreciation for its culturally adapted approach. This findings are consistent with existing literature emphasizing the importance of culturally adapted parenting programs for forcibly displaced parents (Arif & Van Ommen, 2023; Deng & Marlowe, 2013). Participants underscored the challenges associated with resettlement, including acculturation difficulties, changes in family dynamics, and shift in parenting practices. Notably, parents in our study expressed a desire to grasp their new cultural context and better meet their children’s needs. These findings further reinforce that forcibly displaced parents encounter substantial barriers upon resettlement, including acculturation difficulties, loss of social support, and economic stress (Rao et al., 2023; Rosenberg et al., 2022; Sousa et al., 2023). Tailored interventions, such as SafeCare-PCI, provide essential support in addressing these challenges, thereby facilitating the adaptation and well-being of parents in their new environments.

### The Influence of Social Networks and Parental Motivations in Engagement with SafeCare-PCI

Social networks played a critical role in facilitating refugee parents’ engagement with SafeCare-PCI, underscoring the importance of community connections in the resettlement process. These networks remain crucial post-resettlement, as they assist in rebuilding support systems despite disruptions from displacement (Wachter et al., 2022). These networks also facilitate information sharing and “vouching” among displaced parents (Ziersch et al., 2023), which is vital for the uptake of parenting programs as forcibly displaced individuals often rely on family and friends for guidance (Diese et al., 2022). In this study, participants reported that their engagement with SafeCare-PCI was largely driven by recommendations from their social network, particularly as these connections emphasized the program’s potential to enhance understanding and bonding with their children, especially in a new cultural context. These findings are consistent with participants’ expressed motivations to enhance their parenting skills and better address their children’s developmental needs within the context of resettlement.

Displaced parents often struggle with identity issues and unfamiliar cultural norms, (Deng & Marlowe, 2013), which can result in feelings of parenting inefficacy and disconnection (Bergnehr et al., 2018). The SafeCare-PCI program provides an opportunity to mitigate these challenges by improving parenting knowledge and fostering stronger parent–child bonds (Hamari et al., 2022; Kristen et al., 2024).

In addition to the influence of social network, parents in this study received a small monetary incentive to complete SafeCare-PCI sessions as part of the research trial design. Although all participants expressed appreciation for the gift cards, parents across the three cultural groups emphasized that their decision to participate was driven by a deeper interest in the parenting knowledge and skills the program provided, rather than the financial incentive. These findings align with existing literature which suggests that while financial incentives may encourage initial enrollment, they do not necessarily diminish the quality of impact of parenting programs (Dumas et al., 2010; Gross & Bettencourt, 2019; Heinrichs & Jensen-Doss, 2010). In this study, the monetary incentive served as a supplementary benefit, while the parents’ intrinsic motivation to improve their parenting practices and better understand their children’s needs emerged as the primary driving force behind their engagement with SafeCare-PCI. Therefore, while incentives played a role in participant recruitment, they did not overshadow the program’s perceived value in supporting parents’ adjustment to new cultural and parenting norms.

### Cultural Competence and Provider Support in SafeCare-PCI Engagement and Implementation

In this study, refugee parents stressed the importance of flexible, culturally competent providers in facilitating engagement with SafeCare-PCI. Parents values provider who adapted to their needs, demonstrating genuine concern for both their child’s well-being and their unique circumstances. This adaptability, such as adjusting schedules or offering virtual sessions, allowed parents to participate meaningfully in the program, as they felt their personal and familial contexts were understood and respected. Many parents noted that when providers displayed patience and adjusted their approach to accommodate a child’s needs during sessions, it contributed to a supportive learning environment that facilitated deeper engagement with the content.

These findings align with broader literature on the importance of provider flexibility in parenting programs, where both practical and emotional support are essential for successful program engagement and implementation (Foster et al., 2022). Further, research suggests that culturally adapted interventions, when paired with provider flexibility improve both acceptability and effectiveness in teaching new skills (Murphy et al., 2023; Turner et al., 2020). Notably, virtual delivery of parenting programs has been associated with higher participant retention and equivalent skill acquisition as compared to in-person implementation (Self-Brown et al., 2024). This virtual support proved invaluable for many parents, who otherwise might have struggled to navigate the program due to technological or scheduling barriers.

In addition to flexibility, parents in our study highlighted the role of culturally competent providers in strengthening the program’s impact. Providers who were culturally similar or competent played a key role in building trust and enhancing the learning experience, as they could more effectively communicate and related to parents’ concerns. This cultural resonance is crucial in promoting the acceptability and scalability of interventions (Gilliespie et al., 2022; Turner et al., 2020). Parents appreciated when providers spoke their native language and shared similar lived experiences. CHWs in our study and others offer cross-cultural understanding and perform “cultural brokering” (Riza et al., 2020), bridging traditional beliefs with new cultural norms. This task-shifting approach, where CHWs support the integration of culturally appropriate practices in new interventions, increases engagement, effectiveness, and long-term adoption of health interventions (Gonzalez Benson et al., 2024; Greene et al., 2024).

### Impactful Parenting Skills and Strategies Learned from SafeCare-PCI

Parents in our study identified several parenting skills learned through SafeCare-PCI that significantly enhanced interaction with their children, contributing to stronger parent–child bons and more effective behavior management. One key strategy emphasized by parents was the establishment of routines, which played a central role in managing children’s behavior and fostering emotional security. Parents noted that consistent routine, especially around bedtime and daily activities helped children feel more secure and reduce behavioral challenges. These findings echo existing literature which suggested that routines not only support emotional regulation but also promoted healthy lifestyles and improve the overall parent–child relationship (Marsh et al., 2020). Further, routines may mitigate parent–child conflict, alleviate children’s loneliness and depression, and support self-regulation, which is particularly critical for children experiencing the stress of displacement (Bater and Jordan, 2017; Liu et al., 2021).

Parents also highlighted that offering their children choices in daily activities increased involvement and communication. Allowing children to make decisions about activities, clothing, or toys provided them with a sense of autonomy, which was especially valuable for displaced children whose sense of agency Providing choices fosters a sense of agency can be compromised during migration (Wessells, 2021). This approach not only boosted motivation and enjoyment of daily activities, but also support their social learning, which contributes to cognitive development and future participation in community life (Percy-Smith, 2014; Skene et al., 2022). By giving children more control over their decisions, parents reported feeling more connected to their children’s needs and preferences, which strengthened the parent–child relationships and enhanced their parenting effectiveness.

Additionally, parents found that preparing children about upcoming activities, such as meals or transitions between tasks, helped reduce anxiety and promote smoother interactions. Many parents shared that informing their children about what was to come made the child more mentally prepared and less resistant to changes in routine. This skill mirrors findings from other studies that show how preparing children for events—such as medical procedures or transitions—can have significantly reduce anxiety and increase cooperation (Edwinson, 1992; Månsson, 2023).

Parents across all cultural backgrounds reported that learning these skills helped them better regulate their children’s emotions and fostered a deeper understanding of their children’s preferences and needs. Moreover, these skills facilitated active communication, which has been shown to positively impact children’s cognitive development, particularly in the context of parent–child interaction (Hart & Risley, 1995).

### Evaluating SafeCare-PCI Implementation: Cultural and Linguistic Considerations

The translation quality of SafeCare-PCI materials emerged as a key concern for participants in this study. While most parents reported minimal issues with the translated materials, some noted mistranslations or poor interpretations which could potentially limit program effectiveness. Specifically, Aghan and Burmese mothers noted that certain translations appeared to be generated by automated tools, resulting in awkward or unclear phrasing. Moreover, cultural nuances were sometimes lost in transition, as evidence by one Burmese mother’s observation that the phrasing of “Yes” and “No” questions were perceived as impolite and culturally inappropriate. Although SafeCare-PCI materials were translated and reviewed by native speakers before use, these findings underscore the limitations literal translations which might have led to misinterpretation (Havighurst et al., 2022).

It is important to consider that variations in fluency in both English and the parents’ native languages, as well as differences in dialects and regional idioms could also influence comprehension (Perera et al., 2020). Interviewer debriefing revealed that some participants had limited English proficiency, which may have impacted their ability to fully engage with the content, even when translations were generally accurate. Additionally, these variations in literacy could have influenced their understanding of the materials, with some parents more likely to identify translation issues than others. Despite these linguistic and literacy expressed appreciation for the visual examples included in the materials, which can be especially beneficial for individuals with limited literacy or non-native language skills (Schecter & Otoide, 2010).

Parents also requested more culturally relevant examples of SafeCare skills to better reflect their parenting practices and cultural contexts. As one Congolese mother suggested, incorporating a wider variety of parenting scenarios, such as those related to bathtime and mealtimes, would help her better align her cultural approach to child-rearing to that of the social norms in the U.S. Despite these requests for more tailored content, the visual components of SafeCare-PCI were generally well received.

Overall, while visual components of SafeCare-PCI were valued, feedback on verbal translations was more varied. The request for more culturally adapted examples highlights the need for parenting programs to be tailored to the specific cultural contexts and needs of refugee families (Elliott et al., 2022).

### In-person vs. Virtual SafeCare-PCI Sessions: Parents’ Experiences and Preferences

Participants in our study valued both virtual and in-person formats of SafeCare-PCI, indicating that SafeCare-PCI is effective across both delivery methods. Many parents found the virtual format particularly accessible due to the flexibility it provided in managing home responsibilities and childcare. As one parent noted, virtual session allowed for greater ease in attending while juggling daily tasks, such as taking care of children and managing household duties.

At the same time, one parent suggested that initial in-person group sessions could benefit refugee mothers by providing opportunities for peer learning and child interaction. Such in-person interactions can alleviate parenting stress and isolation among forcibly displaced parents, (Liamputtong et al., 2016; Stewart et al., 2015), as they may help parents rebuild their social networks and gain valuable information (Block et al., 2018; Stewart et al., 2017). These peer groups can foster a sense of community and shared learning, essential for parents navigating challenges of resettlement.

Preference for in-person versus virtual session varied among parents, suggesting that both delivery methods offer distinct benefits. Research also shows that online parenting programs are comparable to in-person ones (Leijten et al., 2024), with some studies showing higher engagement for telehealth interventions (Cai et al., 2024; Grafft et al., 2022; Self-Brown et al., 2024). However, feedback from parents in our study suggested that integrating both virtual and in-person sessions could potentially offer the most flexible and supportive learning environment for refugee families.

### Adapting to U.S. Parenting Norms: SafeCare-PCI’s Role in Supporting Refugee Parents

Forcibly displaced parents, including those in our study, often encounter challenges with parenting post-resettlement (Akesson & Sousa, 2020; Anakwenze et al., 2021; Bryant et al., 2021; Moinolmolki et al., 2021). In this context, SafeCare-PCI offered valuable support, helping parents navigate new child-rearing practices within the U.S. cultural framework. While some parenting practices differed from those in their home countries, many parents found SafeCare-PCI to be a useful resource for learning new strategies that fostered positive relationships with their children. For instance, some parents noted that physical discipline or reliance on informal childcare, such as families or neighbor’s, was more common in their home countries. However, parents in our study expressed an appreciation for the emphasis SafeCare-PCI placed on positive discipline methods, which they found beneficial in their adjustment to life in the U.S. One Afghan mother mentioned that the transition to these new parenting norms could be challenging, particularly depending on the length of residence post-resettlement. Newly resettled parents, often prioritizing urgent needs such as housing, language acquisition, and employment, may find it difficult to fully engage with and adopt these new practices (Cranston et al., 2021; Mangrio et al., 2019). While acculturation levels and familiarity with U.S. parenting practices varied across participants in our study and others (Dahlan et al., 2022; Golub et al., 2018), no evidence exists that parents in our study found the adapted SafeCare materials culturally unacceptable or difficult to understand. Instead, they acknowledged the value of the program in helping them adjust to the cultural expectations of parenting in their new environment.

## Lessons Learned

Key lessons learned from this study highlight several important considerations for implementing culturally adapted parenting programs for refugee families. Although professional translators were used, some participants reported issues with awkward phrasing and culturally inappropriate terms, suggesting that translation quality should be continuously monitored for accuracy and cultural relevance. Regarding delivery methods, both virtual and in-person sessions were valued, with virtual sessions offering flexibility for parents managing household responsibilities, while in-person sessions may provide opportunities for peer learning and social support. A hybrid approach combining both methods may enhance engagement and program effectiveness. Culturally competent provider support was also crucial, as providers who understood parents’ cultural backgrounds and spoke their native language helped foster trust and engagement. Typically, SafeCare is implemented using a rigorous coaching model with SafeCare Trainers reviewing recordings of sessions to ensure fidelity to the model (Whitaker et al., 2024). Here, this was not possible as SafeCare experts did not have the linguistic capacity to understand the sessions. Despite the use of financial incentives, parents’ participation was largely driven by intrinsic motivations to improve their parenting skills and support their children’s well-being. Lastly, while acculturation challenges were evident, SafeCare-PCI effectively helped parents navigate U.S. parenting norms, highlighting the importance of addressing cultural shifts while respecting diverse parenting practices.

## Limitations

This study contributes to the limited evidence on culturally adapted parenting programs but has several limitations. The small sample size restricts the generalizability of our findings, and our focus on three ethnic communities—due to resource constraints—may not address the needs of other groups. Additionally, the generalizability of these findings may be impacted by the delivery of the program; virtual, in-person, mandatory, voluntary, etc. The adaptation process yielded a single adapted curriculum for these communities rather than separate culturally specific curricula (Self-Brown et al., 2022), potentially limiting its relevance for others. Further, only including English-speaking participants may have excluded insights on SafeCare-PCI experiences from non-English speakers. This may have introduced bias by disproportionately excluding parents who are less acculturated or more linguistically isolated, potentially limiting the study’s ability to identify cultural or communication barriers. Future research should incorporate multilingual data collection strategies to improve inclusion and representation. The study’s focus on parents who completed all sessions of SafeCare PCI also excludes perspectives of parents who refused or dropped out, which may affect understanding of the program’s relevance for these individuals. This introduces the possibility of selection bias, as participants who completed the program may have had more positive experiences than those who disengaged. As a result, the findings may overrepresent favorable views of the program and underreport challenges or negative experiences. Finally, this analysis presents only qualitative findings. Quantitative measures of skill improvement, parenting stress, child behavior, and support are being collected and will be reported separately. However, the current reliance on self-reported data may be subject to social desirability or selective recall bias. While interviews provided in-depth insight, the absence of triangulation with observational or quantitative data limits the strength of claims about the program’s effectiveness. Future studies should consider integrating multiple data sources to enhance methodological rigor and validity.

## Implications and Conclusions

Our study indicates that refugee parents responded positively to the cultural adaptation of the SafeCare-PCI program, although there are recommendations for future practice. Feedback was overwhelmingly positive for CHWs, with minimal comments on agency staff, possibly due to cultural dissonance as CHWs were members of the community to which the parent belonged, whereas not all agency staff were. Future studies should explore differences in SafeCare-PCI outcomes between implementation methods and adjust delivery accordingly. Many parents reported being recruited for SafeCare-PCI through community networks, highlighting the importance of these channels in information and experience sharing among forcibly displaced individuals. Future iterations of SafeCare-PCI could benefit from enhancing these networks to improve recruitment and engagement and from adjusting the curriculum to accommodate group interaction for parents to connect, share experiences, and learn collaboratively.

Participation in this SafeCare-PCI program was via a research project, where all participation was voluntary. Some SafeCare implementations across the U.S. serve parents who are mandated within child protection systems because of reported or substantiated maltreatment. Engagement, learning, and acceptance may differ between mandatory and voluntary participants, warranting further investigation into how these differences impact parenting practices and program participation.

Some parents mentioned translation issues. Notably, while parents in this study identified with one of three ethnic communities, their cultural backgrounds might differ. Although SafeCare-PCI was offered in five different languages, these may not always align with participants’ native languages, and regional or cultural language variations could affect interpretation. Future iterations of the SafeCare curriculum should address these cultural nuances in translation. Additionally, variations in English fluency and length of U.S. residency in our sample may have impacted engagement with the materials. Future research should investigate how language fluency and acculturation levels impact SafeCare results.

The use of technology presented barriers to engagement for some parents, though this was largely mitigated by competent and culturally sensitive CHWs. Digital literacy levels varied among parents and could have affected participant engagement and the overall success of the programs. Future iterations should include guidance for parents on how to use technology effectively to better participation.

Previous studies indicate that culturally adapted parenting programs can positively change parenting practices among forcibly displaced parents. This study confirms refugee parents’ acceptance of a culturally tailored positive parenting program and underscores the value of such support for parents adjusting to a new cultural context. Adapting parenting programs to align with different cultural norms facilitates integration into new communities and reinforces the foundational principle highlighted in socioecological theories regarding the role of parenting in child development. Further, positive parenting can help forcibly displaced parents build deeper relationships with their children, significantly benefiting their development. Despite limitations, this study emphasizes the importance of culturally adapted parenting programs for supporting forcibly displaced families upon resettlement and highlights the need for increased accessibility.

## Supplementary Information

Below is the link to the electronic supplementary material.

Supplementary file1 (DOCX 18 KB)

## Data Availability

The data generated and/or analyzed during this study are not publicly available due to privacy and confidentiality concerns related to study participants. In accordance with ethical guidelines and data protection regulations, the data are stored securely and can only be accessed by authorized personnel. For any inquiries regarding data access or to request further information, please contact the first author.
