# Supplementary material for: Navigating Cultural Adaptation: Refugee Parents’ Perspectives on the SafeCare Parenting Program
Source: J Int Migr Integr. 2025 Jul 28;27(1):141–68. doi: 10.1007/s12134-025-01299-1 (PMC13046571; doi:10.1007/s12134-025-01299-1)
Supplement: Supplementary file 1 — Supplementary file1 (DOCX 18 KB) [file 12134_2025_1299_MOESM1_ESM.docx]

**Appendix A**

Interview Guide for Parents That Completed SafeCare-PCI

1. **[Recruitment]** Can you tell me what you first heard about SafeCare?
   1. Where did you first hear about this program and what did you hear?
   2. What did you hope to get out of enrolling in the program?
2. **[Benefits]** What most interested you about the program? For example….
   1. **Probes:**
      1. Were you looking forward to helping to manage your child(ren)? *If affirmative response:* What kind of help were you hoping to get?
      2. Were you interested in having someone that you could talk to about your children? *If an affirmative response:* Can you tell me more?
      3. Were you hoping the program might help you personally? *If affirmative response:* How were you hoping the program would help you specifically?
      4. How interested were you in receiving the gift cards?
3. **[Benefits]** Can you describe your overall experience with SafeCare?
   1. **[SC Skill use]** Can you tell me what things you learned in SafeCare that you were not doing before you had the program?
   2. **[SC Skill Use]** What was most helpful about SafeCare?
      1. **Probes:**
         1. For example, how did SafeCare help you have more positive interactions with your child?
         2. How did SafeCare help you to create a routine for your child?
4. **[Satisfaction]** What are the things you liked about the program?
   1. **Probes:**
      1. Can you give me a specific example?
      2. Were there any specific skills or activities that you especially liked?
5. **[Satisfaction]** What are the things you disliked about the program?
   1. **Probes:**
      1. Were there specific skills that were taught that were not helpful? *If affirmative response:* What skills were they? How come they are not helpful?
      2. Was the number of sessions or the timing of sessions inconvenient? *If affirmative response:* What would work better?
6. **[Reasons to join/stay]** If you were talking to a friend about the program, what would you say?
   1. **Probes:**
      1. Would you recommend it to them, Why/Why not?
      2. What if there were no gift cards?
7. **[Provider Satisfaction]** We are interested to hear how families got along with their providers. Do you feel like your provider understood and respected you and your family? (Probe: What I mean is how you feel, how you and your family act toward each other, and your roles and responsibilities?)
   1. *If affirmative response:* How did your provider show that they understood and respected you and your family? Can you give me a specific example?
   2. *If negative response:* What are some things that made you feel that your provider did not understand or respect you or your family? Can you give any specific examples? Just to reassure you, nothing you would tell us will get back to your provider; we just want to try to improve the program.
   3. **[Provider Satisfaction]** Can you describe any ways that your provider helped you participate in the program?
      1. **Probes:**
         1. For example…
            1. Did they help with technology? *If an affirmative response:* How so?
            2. Did they meet you in certain places? *If affirmative response*: Where did you meet? How did meeting at this (these) place(s) work out?
   4. **[Provider Satisfaction]** Is there anything you would change about your provider?
8. **[Culturally appropriate]** Next, we are interested to know if the things you were taught during SafeCare were in line with your cultural values. Were the things you were taught very different or similar to how your culture teaches you how to parent? How so?
   1. Were the SafeCare materials you used translated into [*reference native language*]?
      1. *If affirmative response:* To what extent were the translations correct? (Probe: How might the translations be improved upon?)
      2. *If negative response:* Did you understand the materials, easily? (Probe: What made it hard to understand the materials?)
      3. **[Culturally appropriate]** What changes would you recommend to the materials?
